# Relationship between the Microstructure and Performance of Graphene/Polyethylene Composites Investigated by Positron Annihilation Lifetime Spectroscopy

**DOI:** 10.3390/nano11112990

**Published:** 2021-11-06

**Authors:** Xiaobing Han, Tao Chen, Yuan Zhao, Jie Gao, Yanan Sang, Houhua Xiong, Zhiyuan Chen

**Affiliations:** Hubei Key Laboratory of Radiation Chemistry and Functional Materials, School of Nuclear Technology and Chemistry & Biology, Hubei University of Science and Technology, Xianning 437100, China; hanxiaobing@hbust.edu.cn (X.H.); taochen518@163.com (T.C.); zhyf308@hbust.edu.cn (Y.Z.); sangyanan2021@163.com (Y.S.); xionghouhua@hbust.edu.cn (H.X.)

**Keywords:** graphene, polyethylene, positron annihilation, microstructure, property

## Abstract

The quantitative characterization of microstructure is most desirable for the establishment of structure-property relationships in polymer nanocomposites. In this work, the effects of graphene on the microstructure, mechanical, electrical, and thermal properties of the obtained graphene/polyethylene (PE) composites were investigated. In order to reveal the structure-performance relationship of graphene/PE composites, especially for the effects of the relative free volume fraction (*f_r_*) and interfacial interaction intensity (*β*), positron annihilation lifetime spectroscopy (PALS) was employed for its quantitative description. The relative free volume fraction *f_r_* gives a good explanation of the variation for surface resistivity, melting temperature, and thermal stability, and the variation of tensile strength and thermal conductivity agree well with the results of interfacial interaction intensity *β*. The results showed that *f_r_* and *β* have a significant effect on the properties of the obtained graphene/PE composites, and the effect on the properties was revealed.

## 1. Introduction

Polyethylene (PE) is one of the most important commercial polymer materials. In the PE family, low density PE has a wide application in our daily life due to its low cost and good flexibility [1,2]. When carbon-based nanofillers are incorporated, PE composites with improved mechanical, electrical, and thermal properties can be produced [3,4]. These PE nanocomposites have potential for applications such as static charge dissipative materials, semiconductor layers, thermal management materials, and gas barrier materials [5,6,7,8,9,10]. Within these carbon-based nanofillers, graphene has grown most rapidly due to its excellent mechanical, electrical, thermal, and barrier properties and huge surface area [11,12,13,14,15,16,17,18,19].

The key factor that influences the properties of graphene/polymer composites is the microstructure, including the relative free volume fraction (*f_r_*) of the obtained composites, and the interfacial interaction (*β*) between graphene and the polymer matrix. As *β* determines the dispersion state of graphene, it has a dramatic influence on graphene’s stress dispersion and phonon scattering. In addition, the *f_r_* determines the transport performance, such as electrical conductivity, thermal stability, and gas/liquid barrier properties [19,20,21,22]. Although significant progress has been made in the development of graphene/PE composites [23,24,25,26,27,28], and high-performance composites with excellent mechanical, electrical, and thermal properties have been obtained, there are few reports about the microstructure (*f_r_* and *β*), and the correlation between these parameters and properties for graphene/PE nanocomposites.

Positron annihilation lifetime spectroscopy (PALS) is a useful tool in determining the atomic scale defects and free volume hole for a wide variety of materials [20,29]. The positron produced with a radioactive ^22^Na is diffused into the polymer, then gets thermalized rapidly with losing energy. The thermalized positron is injected into the material, annihilating directly with free electron or indirectly by forming positronium (P). Positrons are preferentially localized in the cavity of the free volume hole. According to the free volume hole model, the pick-off annihilation lifetime *τ*_3_ is related to the cavity size. When the cavity size of the free volume hole increases, the opportunity of electron seen by *o*-Ps decreases, leading the lifetime of *o*-Ps to become longer [30].

Recently, the PALS technique can not only be used for the probe of the free volume hole, but also for the characterization of the interfacial interaction. The second lifetime *τ*_2_ is assigned to the positron annihilation in various vacancies [31]. The change of the second lifetime intensity (*I*_2_) is related to the interfacial interaction in the polymer composites. According to the simple mixture rule, if there is no interfacial interaction between polymer matrix and filler, *I*_2_ only comes from the annihilation in the matrix and filler, respectively. In this case, *I*_2_ is the linear correlation with the content of filler. Actually, the interfacial interaction always exists in polymer composites, thus deviations occurred between the theoretical results and the experimental results [19,32]. Consequently, the interaction parameter *β* was introduced to investigate the interface between the polymer matrix and the filler [33,34]. This provides a new approach for the construction of the relationship between microstructure and performance, but the utilization of PALS in graphene/PE composites has rarely been reported. 

In this work, the PE incorporated with different contents of graphene were prepared by premixing followed by hot pressing. Firstly, the interfacial interaction of the obtained graphene/PE composites was qualitatively characterized with traditional infrared spectroscopy. In order to give a deep insight into the microstructure, PALS was used to determine the free volume hole characteristics and interfacial interaction intensity of graphene/PE composites. The results showed that the *f_r_* and *β* had a significant effect on the mechanical, electrical, and thermal properties of the obtained graphene/PE composites, and their effect on the properties was revealed.

## 2. Materials and Methods

### 2.1. Materials

Graphene was purchased from Suzhou TanFeng Graphene Tech Co., Ltd. (Suzhou, China). Low density polyethylene (Mn = 122,000 g/mol, PDI = 1.5) was purchased from Aladdin Chemical Reagent Co., Ltd. (Shanghai, China). n-Hexane was obtained from Sinopharm Chemical Reagent Co., Ltd. (Shanghai, China) without purification.

### 2.2. Preparation of Graphene/Polyethylene Composites

Graphene/PE composites with different graphene concentration (0~2.0 wt%) were prepared by premixing followed by hot pressing. Following typical procedure, the graphene was dispersed into 40 mL n-Hexane using a bath sonicator for 1 h. Then, 8 g of PE powder was added into the dispersion with vigorous stirring for 2 h and the mixture was sonicated for 1 h. The obtained mixture was centrifuged at 4000 r/min for 0.5 h and the precipitate was dried at 60 °C under vacuum to obtain a constant weight. Finally, the obtained mixture was pressed at 140 °C, 20 MPa for 10 min with a d = 0.5 mm steel mould.

### 2.3. Characterization

Fourier transform infrared (FTIR) spectra were recorded on an Avatar 360 Nicolet instrument (Thermo Fisher Scientific, Shanghai, China) by measuring the powder mixture. The mechanical properties of the graphene/PE composites were measured by a tensile testing machine (Shimadzu AG-IC, Zhujin Analytic Instruments Co., Ltd., Shanghai, China). Five pieces of each sample were tested to obtain average values. The surface resistance of the obtained composites was measured with an ultra-high resistance micro-current tester (ST2643, Suzhou Jingge Electronic Co., Ltd., Suzhou, China). The thermal conductivity of the composites was measured using thermal conductivity test equipment (DRE-2C, Xiangtan Instrument Co., Ltd., Xiangtan, China). The Differential scanning calorimeter (DSC) curves were recorded with a DSC-200-F3 (Netzsch, Selb, Germany) from 30 to 160 °C, with a heating rate of 10 °C/min. Thermogravimeter (TG) analysis was conducted with a TG-209-F3 (Netzsch, Selb, Germany) under nitrogen atmosphere with a heating rate of 10 °C/min.

## 3. Results

### 3.1. Interfacial Interaction between Graphene and PE

**Figure 1:**
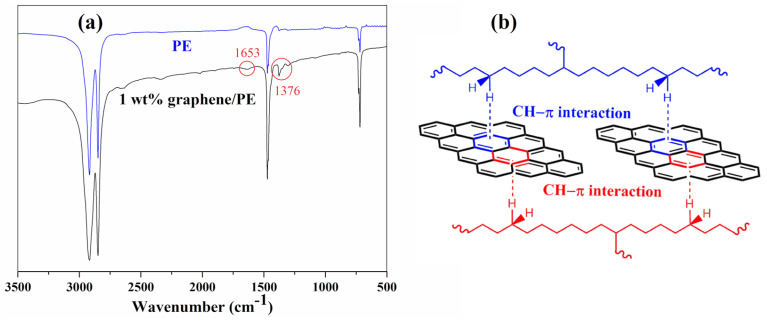
(**a**) FTIR spectra and (**b**) CH–π interactions between graphene and PE.

To demonstrate the chemical structure of pure PE and reveal the interfacial interaction between PE and graphene, the FTIR spectra of PE and 1 wt% graphene/PE composites were shown in Figure 1a. Three sets of signals—the C-H stretching vibration signals at 2920 and 2848 cm^−^^1^, the C-H bending vibration signal at 1471 cm^−1^, and the C-H rocking vibration signal at 721 cm^−^^1^—are present in the spectrum of PE [35]. All sets of signals are also present in the 1 wt% graphene/PE composites, though there is no appreciable difference between the pure PE and the composite for the C-H stretching vibration and rocking vibration signals. However, there is a distinct difference in the C-H bending vibration signal between the two samples. Unsurprisingly, there are two new peaks at 1653 and 1376 cm^−1^ observed in the composite, and this change in the C-H bending vibration signal is evidence supporting the occurrence of CH–π interactions between PE and graphene sheets [17,35]. According to the results of the FITR spectra, an illustration of the CH–π interactions was provided in Figure 1b. Although the type of interfacial interaction can be revealed with FTIR spectra, the information of interfacial interactions intensity cannot be obtained. 

### 3.2. PALS Analysis of Graphene/PE Composites

To quantitatively reveal the effects of graphene on the microstructure of graphene/PE composites, the PALS was recorded. The PALS was carried out by a conventional fast–fast coincidence spectrometer at room temperature. The positron source ^22^Na (15 μCi) was sealed in a 7 μm thick Kapton foil and sandwiched between two identical pieces of sample for measurement. One million counts were recorded for each spectrum, which was resolved into three components by a PATFIT fitting program. The long lifetime component *τ*_3_ was used to calculate the volume of free volume hole (*V_f_*) according to the following equations:

(1) $$\tau_{3}=\frac{1}{2}{\left[1-\frac{R}{R+\Delta R}+\frac{1}{2\pi }\sin\left(\frac{2\pi R}{R+\Delta R}\right)\right]}^{-1}$$

(2) $$V_{f}=\frac{4}{3}\pi R^{2}$$

*R* represents the radius of the cavity, and Δ*R* = 1.656 Å [20,33]. The intensity of *o*-Ps (*I*_3_) is related to the concentration of the free volume hole. The fraction (*f*) of free volume hole was calculated as:

*f* = *CV_f_I*_3_ (3)

*C* can be approximately regarded as a constant [20,36], with a value of 0.0018 Å^−3^; the relative free volume fraction (*f_r_*) is defined as:

*f_r_* = *V_f_I*_3_ (4)

**Figure 2:**
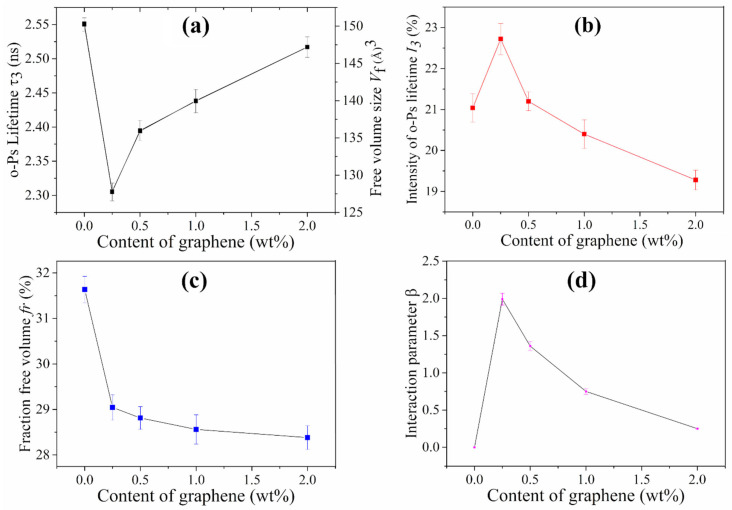
(**a**) Long lifetime (*τ*_3_) and free volume size (*V_f_*); (**b**) long lifetime intensity (*I*_3_); (**c**) relative free volume fraction (*f_r_*); (**d**) interaction parameter (*β*) of the graphene/PE composites.

The long annihilation lifetime (*τ*_3_), free volume hole size (*V_f_*), the formation probability of *o-Ps* (*I*_3_) and the relative fraction (*f_r_*) of the free volume hole as a function of graphene content were shown in Figure 2a–c, respectively. As shown in Figure 2a, all the composites have a lower *τ*_3_ than that of pure PE, suggesting that the obtained composites have a lower mean size of free volume hole, which is consistent with the results of PVA/rGO composites [33]. The smallest *τ*_3_ and *V_f_* for the composites were observed containing 0.25 wt% graphene. This can be ascribed to the highly exfoliated and well dispersed graphene at low content, where the large surface area provided by the graphene is beneficial to the formation of CH-π interaction (Figure 1b), restricting the mobility of PE chains and resulting in a decrease of *τ*_3_ and *V_f_*. In addition, *τ*_3_ and *V_f_* showed an increase trend with the increase of graphene content; this is ascribed to the agglomeration of graphene sheets, in which weak interactions between stacked graphene and PE chains has a little limitation on the chain mobility, causing a larger *τ*_3_ and *V_f_* [19,33].

As show in Figure 2b, the variation of *o*-Ps lifetime intensity (*I*_3_) is opposite to the *o*-Ps lifetime, which has also been observed in the PVA/rGO composites reported by Wang and co-workers [33]. This can be explained as the reason that the graphene sheets can disrupt the PE molecular chain configuration, leading to the increase in the free volume hole concentration. The well dispersed graphene sheets can disarrange the molecular configuration, resulting in the morphological change of PE chains and increase of the free volume hole concentration and *I*_3_. However, with a further increase of graphene content, this disruption of graphene gets weak due to the agglomeration of graphene, which results in the decrease of free volume hole concentration and *I*_3_ [19,33].

Based on the analysis of the free volume hole size and the free volume hole concentration, the relative free volume fraction (*f_r_*) is present in Figure 2c showing that the *f_r_* decreases with the increase of graphene content (31.63% to 28.38%), meaning that graphene sheets limit the moving space of PE chains [37]. These results quantitatively revealed the free volume hole characteristics, which will have a significant effect on the electrical property, melting temperature, and thermal stability of the obtained composites.

The interfacial interaction determines the dispersion state of graphene; thus, it has a dramatic influence on its stress dispersion. For the description of the interfacial interaction, PALS is more useful than FTIR spectra. The FTIR can only determine the type of interfacial interaction (Figure 1), while PALS can provide information about the interfacial interaction intensity [19,33]. As reported in the literature, the newly formed interfacial layer is the main difference between the pure polymer and the polymer/nanofiller composite; parts of positron will be annihilated in the interfacial layer, thus the intensity (*I*_2_) change of the second lifetime can be used to analyse the interfacial interaction intensity in the composites. 

According to the simple mixture rule, if no interfacial interaction exists between the graphene and the PE matrix, *I*_2_ only originated from positron annihilation in graphene and PE, which should be a linear correlation to the content of graphene. Actually, interfacial interaction exists in most polymer composites, and variation with the change of nanofiller content. Consequently, the interfacial interaction parameter (*β*) was introduced to reveal the interaction intensity between graphene and the PE matrix, and the *β* can be calculated according the following equation [19,33,34,36,38,39,40]:

(5) $$I_{2}=I_{2}^{G}W+I_{2}^{P}(1-W)+\beta I_{2}^{G}WI_{2}^{P}(1-W)$$

The superscripts *G* and *P* represent the graphene and PE, respectively; W is the graphene weight fraction; the *I*_2_ of graphene here is 94.78% [36]. The interaction parameter *β* as a function of the graphene content is shown in Figure 2d. It is seen that the composites containing 0.25 wt% graphene have the highest *β* (1.99), indicating the strongest interfacial interaction. With the increase of graphene content, the *β* decreased gradually, which is due to the aggregation and poor dispersion of graphene sheets at high loading [19,37], This is in good agreement with the variation of *V_f_*, which will have a significant effect on the mechanical property and thermal conductivity of the obtained composites.

### 3.3. Mechanical Properties of Graphene/PE Composites

**Figure 3:**
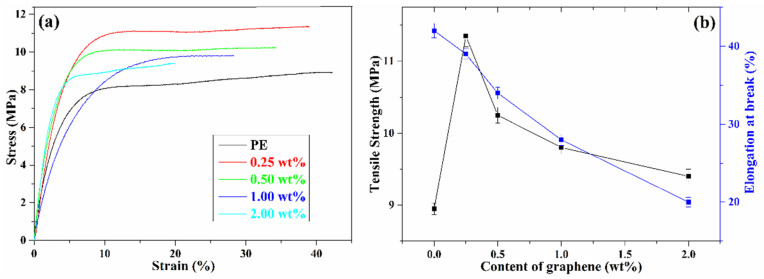
(**a**) Stress–strain curves and (**b**) mechanical properties of graphene/PE composites.

Tensile testing was used to evaluate the mechanical properties of pure PE and the graphene/PE composites. The stress–strain behaviour of all samples is present in Figure 3a. It can be seen that the addition of graphene increases the tensile strength of the PE composites significantly. The tensile strength and elongation at break are also plotted against graphene content in Figure 3b. Compared with pure PE, the tensile strength of the composites increased at low contents of graphene; the tensile strength of the 0.25 wt% graphene/PE composites increased from 8.95 to 11.35 MPa, with an increase ratio of 26.8%. The value of the tensile strength decreased when the graphene content exceeded 0.25 wt%, while still remaining higher than that of PE [27]. This may be assigned to the different dispersed states of graphene, and the different interfacial interactions between graphene and the PE matrix. Graphene sheets were highly exfoliated at low content and exhibit strong CH–π interactions with the PE chain; this interfacial interaction can be destroyed as a sacrificial bond in the stretching, leading to the effective dissipation of energy. In addition, the aggregation of graphene at high content exhibits weak interfacial interaction, leading to a poor dissipation of stress [41,42]. This result is consistent with the change of interaction intensity *β* against graphene content (Figure 2d), because the interfacial interaction can transfer stress from the polymer matrix to the nanofiller. A similar phenomenon has also been observed in GO/WPU composites [19], which demonstrated that the relationship between interfacial interactions and mechanical properties can be established using the PALS technique. The elongation at break declines significantly with increasing graphene content, which can be ascribed to the decrease of flexibility of the polymer chain. As mentioned in the PALS analysis, the *f_r_* decreases with the increase of graphene content (Figure 2c), meaning that the graphene sheets decrease the moving space of the PE chains; thus the flexibility of the PE chain is decreased by the increase in graphene content [27].

### 3.4. Surface Resistivity of Graphene/PE Composites

**Figure 4:**
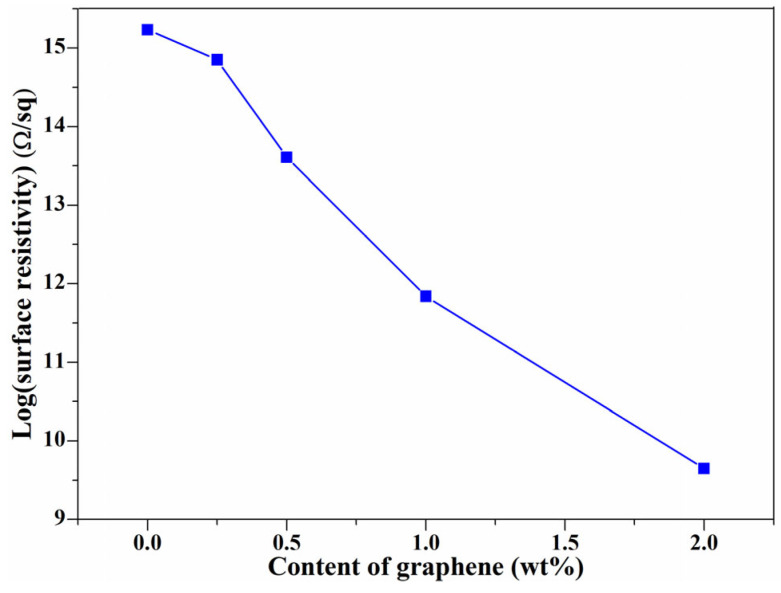
Surface resistivity of PE and graphene/PE composites.

According to electrical percolation theory, graphene sheets provide percolated pathways for electron transfer, which impart electrical conductivity to the composites. However, the improved efficiency of graphene sheets dramatically depends on the relative free volume fraction (*f_r_*) of the obtained composites [34,43]. The dependence of surface resistivity on graphene sheets loading is present in Figure 4; all of the composites have an improved conductive property [23]. As the graphene content increases, the surface resistivity of the composite decreases gradually, which is consistent with the variation of the relative free volume fraction with graphene content (Figure 2c). As the decreased *f_r_* can produce a high-density interfacial region, restricting the mobility and relaxation of PE chains, which is of benefit for the formation of a conductive network, thus lower surface resistivity composites were obtained with a lower *f_r_*. Similar phenomena have also been observed in the PC/rGO composites [34] and graphene/NR composites [43], which revealed that the relationship between the relative free volume fraction and the electrical properties can be established using the PALS technique. 

### 3.5. Thermal Properties of Graphene/PE Composites

**Figure 5:**
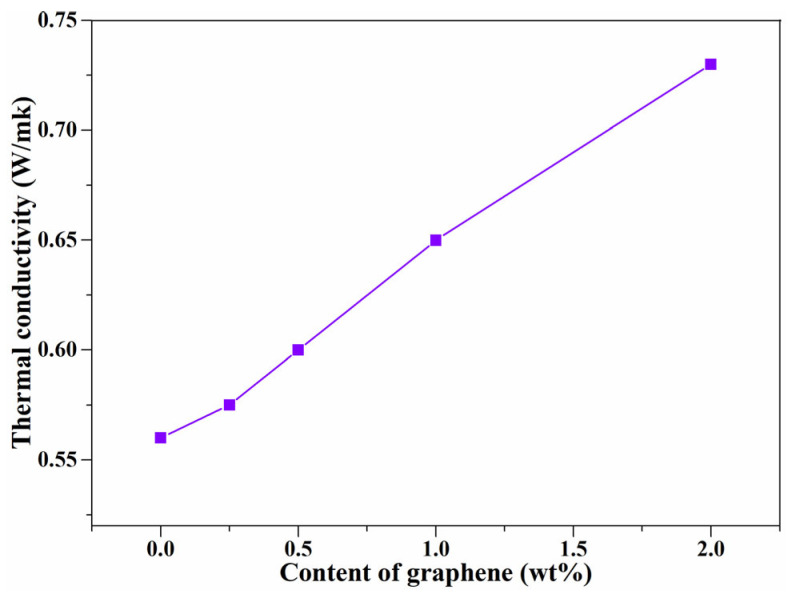
Thermal conductivity of PE and graphene/PE composites.

Thermally conductive polymer composites are attracting considerable attention, especially in recent years, because increasingly, more powerful electronics are being developed [6,44]. The thermal conductivity values of pure PE and PE composites are summarized in Figure 5; the unfilled PE has the lowest value which is 0.56 W/m·K. The thermal conductivity of the graphene/PE composites increased with the increase of graphene content, the highest increase with respect to the pure PE was for the 2.0 wt% graphene/PE composite and was 34%, which can be ascribed to the high thermal conductivity of graphene [26,27]. When the content of graphene is 0.25 wt%, the thermal conductivity of the composites deviates from linear growth, which is due to the strongest CH–π interaction between graphene and PE and because the interfacial interaction provides more sites which can scatter phonons and damp the vibration amplitude at the interface, inducing a higher thermal resistance. With increasing graphene content, the graphene agglomerates and the interfacial interaction-induced thermal resistance is limited [33,45]. This result is consistent with the variation of interaction intensity *β* against graphene content (Figure 2d), because the thermal conductivity is related to the interaction, which can scatter phonons and damp the vibration amplitude at the interface. A similar phenomenon has also been observed in PVA/rGO composites [33], which demonstrated that the relationship between interfacial interaction and thermal conductivity can be established using the PALS technique.

**Figure 6:**
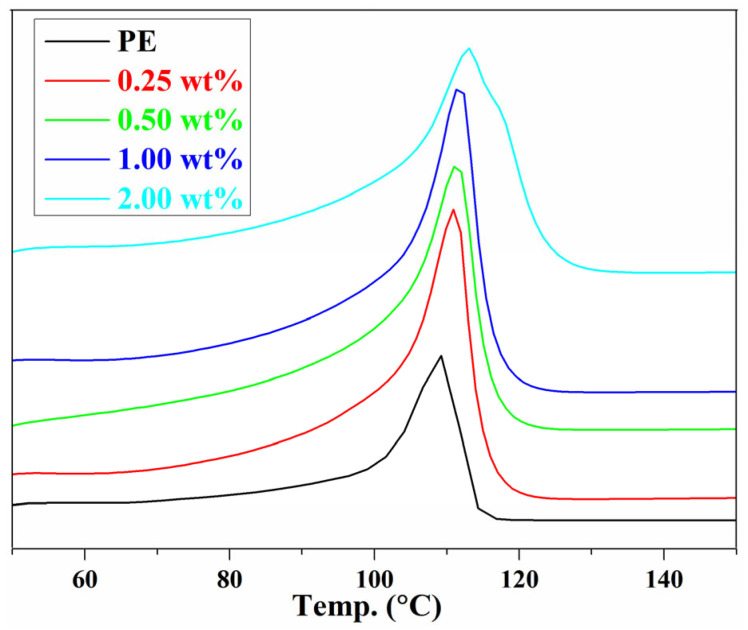
DSC curves of PE and graphene/PE composites.

A differential scanning calorimeter was used for the melting temperature (*T_m_*) characterization of PE and graphene/PE composites (Figure 6). For all samples, only one melting endothermic peak could be observed. The *T_m_* of pure PE is 109 °C, and the melting temperature does not change significantly after incorporating graphene sheets [28]. The melting temperature of the graphene/PE composites increased with the increase of graphene content; the highest increase with respect to the pure PE was for the 2.0 wt% graphene/PE composite and was 3 °C [8]. This result is consistent with the variation of relative free volume fraction *f_r_* with graphene content (Figure 2c), because the *T_m_* is related to the free volume fraction. The lower the *f_r_*, the more difficult it is for the movement of the polymer chain, leading to a higher *T_m_*. A similar phenomenon has also been observed in the GO/WPU composites [19], which revealed that the relationship between relative free volume fraction and melting temperature can be established with PALS technique. 

**Figure 7:**
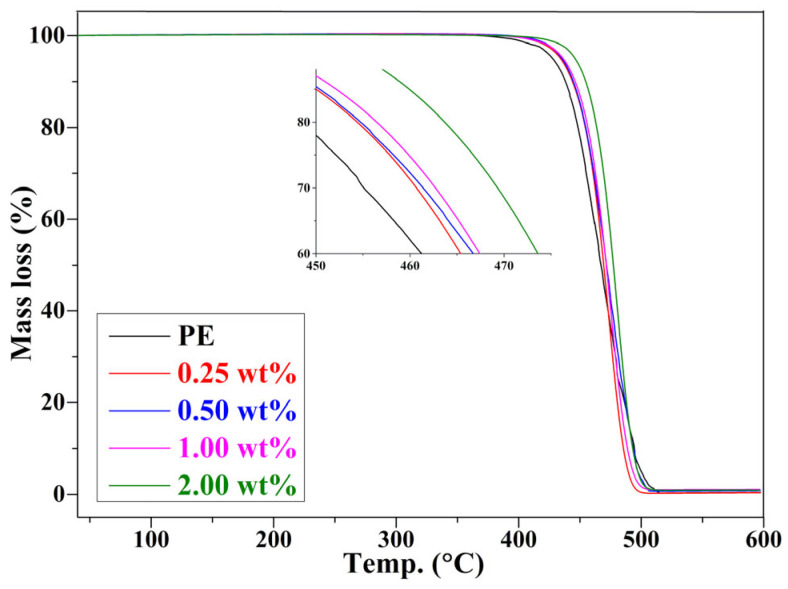
TG curves of PE and graphene/PE composites.

Increased thermal stability is typical for polymer-layered nanocomposites, usually attributed to the heat and mass barrier effects of layered nanocompounds, which delay the diffusion of heat and pyrolysis products. The thermal stability of graphene/PE composites is present in Figure 7. The pure PE shows a fast degradation at a temperature range of 400–500 °C, and almost completely decomposed at 500 °C [25]. It is found that all the composites present similar degradation behaviour to pristine PE, and the composites have an improved thermal stability compared to pure PE. As the graphene content increases, the 10% weight loss temperatures are 436, 441, 445, 448, and 455 °C [28]. This improvement of thermal stability can be ascribed to the tortuous path effect, which formed between graphene and PE through CH-π interaction [17,29]. The thermal stability increased with the increase of graphene content can be ascribed to the effective obstruct towards low molecules from degraded PE, and the shield function to the heat [46]. This result is consistent with the variation of the relative free volume fraction *f_r_* with the graphene content (Figure 2c), because the thermal stability is related to the free volume fraction. The lower the *f_r_*, more difficult it is for the transfer of heat and the diffusion of pyrolysis products, leading to improved thermal stability. This demonstrates that the relationship between the free volume fraction and thermal stability can be established using the PALS technique.

## 4. Conclusions

In summary, the graphene/PE composites were prepared by premixing followed by hot pressing; the microstructure of the obtained composites was determined simultaneously by using the qualitative (FTIR) and quantitative (PALS) methods. Enhanced properties were obtained for the graphene/PE composites, and the structure–performance relationship was established through the quantitative characterization of microstructure (*f_r_* and *β*). The results of FTIR absorption spectra revealed that the CH-π interaction between PE and graphene can be formed in the processing. The CH-π interaction can serve as a sacrificial bond to dissipate stress, leading to the interaction intensity *β* having the same trend as the tensile strength. The highest values of *β* (1.99) and tensile strength (11.35 MPa) were found for the composites containing 0.25 wt% graphene. The decreased *f_r_* is beneficial for the formation of a conductive network, thus the lowest log(surface resistivity) (9.65) was obtained for those composites possessing the lowest *f_r_* (28.38%). The strong interaction intensity *β* for the 0.25 wt% graphene/PE composites can scatter phonons and damp the vibration amplitude, inducing a higher thermal resistance. As the content of graphene increased from 0 to 2.0 wt%, the *f_r_* decreased from 31.63% to 28.38%, making movement of the polymer chain more difficult. This leads to an increase of *T_m_* from 109 °C to 112 °C. As the low *f_r_* can prevent the heat transfer and the diffusion of pyrolysis products, the greatest thermal stability was obtained for the composites possessing the lowest *f_r_* (2.0 wt% graphene/PE). These results give deep insight into the relationship between structure and performance for polymer composites; future work will focus on the establishment of quantitative relationships.

## Data Availability

Data is contained within the article.
